# Relationship Between Quality Perception and Patient Expectations with Patient Demographic Structure in Health Care Institutions and Organizations: Atatürk University Faculty of Dentistry Example

**DOI:** 10.5152/eurasianjmed.2025.23164

**Published:** 2025-03-26

**Authors:** Doğan Durna, Özkan Demirtaş, Nurhan Bayındır Durna, Vecihi Yiğit

**Affiliations:** 1 Department of Oral & Dentomaxillofacial Radiology, Atatürk University Faculty of Dentistry, Erzurum, Türkiye; 2 Institute of Science and Technology, Atatürk University, Erzurum, Türkiye; 3 Department of Orthodonty, Atatürk University Faculty of Dentistry, Erzurum, Türkiye; 4 Department of Industrial Engineering, Atatürk University Faculty of Engineering, Erzurum, Türkiye

**Keywords:** Population health, health services research, socioeconomic factors, personal satisfaction

## Abstract

**Background:**

To reveal whether the quality perceptions service expectations of patients and their relatives who come to health institutions for examination and treatment differ according to their demographic structures.

**Methods:**

The data collection tool used was the Patient Satisfaction Survey. The sample of the study consisted of 428 patients aged 21 and over 21 years who applied to Atatürk University Faculty of Dentistry for dental examinations between April 1, 2022 and July 31, 2022. The descriptive scanning method, one of the quantitative research methods, was used in the study. Survey analyzes were evaluated using scientific statistical analysis techniques described in the literature, and IBM Statistical Package for the Social Sciences (IBM SPSS Corp.; Armonk, NY, USA) Statistics 20 software was used.

**Results:**

Since the normal distribution condition was not met in normality tests, nonparametric tests were performed. Nonparametric tests revealed that patients’ perceptions of quality and expectations did not differ according to gender, education, or marital status. On the other hand, patients’ perceptions of quality and expectations differed according to age, profession, and monthly income.

**Conclusion:**

As a result of the statistical evaluation of the survey results, it was revealed that the quality perception and patient expectations in health institutions and organizations vary according to the demographic structures of the patients such as gender, age, education level, and monthly income.

**Main Points**

- The quality perception and service expectations of the patients participating in the survey do not differ according to their gender.
- The quality perception and service expectations of the patients participating in the survey vary according to age groups.
- The quality perception and service expectations of the patients participating in the survey do not differ according to their educational level.
- The quality perception and service expectations of the patients participating in the survey vary according to the professional groups.
- The quality perception and service expectations of the patients participating in the survey vary according to monthly income groups.

## Introductıon

Public and university hospitals, whose primary purpose is to diagnose human diseases and provide services for the treatment of these diseases, fulfill their public responsibilities without making a profit and provide health services in line with this purpose. Although public hospitals have been providing medical services for many years, significant changes have occurred in the field of medical services in our country, as well as all over the world, and private health institutions have also taken their place in this field. In this field, commercial gain and health services go hand in hand and seem to continue to do so.

Since the main purpose of quality research in both manufacturing and service sectors is to achieve maximum efficiency at minimum cost, companies try to achieve greater efficiency through quality improvement research.

Health services, which were socialized in the 1960s, began to weaken in the face of liberal movements that began to develop in the 1980s and were forced to change. The 2003 Health Transformation Program, in addition to taking the country’s health system to a completely different position, was also the first result of the process of change in the perception of health.^1^

As private healthcare providers compete with each other commercially, these institutions have begun to focus on quality, thinking about how to provide better service to patients, rather than just examining and treating them. Public hospitals have also joined this competitive environment and have made efforts to ensure that patients feel the treatment, other social expectations, and psychological comfort.

The “Quality and Accreditation in Healthcare Institutions” research, which is 1 of the 8 main components of the Health Transformation Program and aims to provide quality healthcare services, was implemented institutionally and systematically by our Ministry in 2005.^2^

If healthcare institutions lag behind in their efforts to improve the quality of their healthcare services and cannot meet patient expectations in the future, they will not be able to compete with institutions and organizations that attach great importance to this field and develop and implement service quality criteria, and they will have difficulty maintaining their existence in the social arena.

Improving the quality of healthcare services will provide the highest level of benefit for patients. When creating satisfaction-based and problem-solving focused healthcare services become a top priority, patients will be sure that they are getting the full value of their choices. Such a belief increases patients’ trust in healthcare institutions.^3^

Since the main purpose of research on the quality of care and treatment in health institutions and organizations is to increase patient satisfaction, surveys are conducted as evaluation and development criteria regarding these facts. Survey results are examined using scientific statistical methods, and the results are interpreted. Based on the obtained data, conclusions regarding improvement and correction are reached.

Many studies have been conducted on this subject in the world and in our country, and positive developments have been reported as a result of the reflection of these studies into practice.

According to Kajral,^4^ in the first approach, researchers evaluate quality in 3 dimensions. These are:

- Physical quality,
- interaction quality, and
- business quality.

There are several main factors that affect patient satisfaction. These factors can depend on the patient, staff, and physical and environmental characteristics. The patient’s sociodemographic characteristics, such as age, education, income, occupation, gender, language, religion, race, and family structure play different roles in patient satisfaction. These criteria vary from person to person and are closely related to the level of satisfaction with health services. Similarly, the staff providing the service and the physical environment can also affect satisfaction with the service.^5^

In this study, which we conducted to determine whether performance standards and patient expectations are perceived differently according to demographic structures at Atatürk University Faculty of Dentistry, quality measurement was used in accordance with the first approach. In this study, survey topics and survey questions were selected in line with this purpose.

## Materials and Methods

This study is a cross-sectional study and does not fall into the category of clinical research. Therefore, informed consent was not obtained from the patients participating in the survey. The study was approved by the Ethics Committee of the Faculty of Dentistry of Atatürk University (decision date: September 28, 2023; decision number: 47). The study focuses on patients’ perceptions of quality and service expectations in healthcare and does not include clinical research.

Patients were informed and participated in the survey given in Table 1 on a voluntary basis. In this investigation, the descriptive Survey Design Method was used as a quantitative research method. Descriptive statistics is the process that aims to describe the characteristics of the individuals or objects participating in the research by using the data obtained by observation on a sample or from the whole universe in the accessible situations.^6^

This investigation was a cross-sectional study and did not belong to the clinical research category. Therefore, informed consent was not obtained from the patients participating in this survey. This study was approved by the Local Ethics Committee of our university (decision date: September 28, 2023; decision number: 47). The study focuses on patients’ quality perceptions and service expectations and does not include clinical research.

Patients were informed and participated in the survey given in Table 1 on a voluntary basis. In this investigation, the descriptive Survey Design Method was used as a quantitative research method.

“Descriptive statistics is the process that aims to describe the characteristics of the individuals or objects participating in the research by using the data obtained by observation on a sample or from the whole universe in the accessible situations.”^4^

### Population and Sample of the Study

The study group of this research consisted of 428 patients aged 21 and over 21 years who came to the hospital for examination and treatment between April 1, 2022, and July 31, 2022, selected by a random sampling method. There were no invalid questionnaires in our survey study. The distribution of participants according to demographic characteristics is shown in Table 2.

### Data Collection Tools

The “Patient Satisfaction Questionnaire” was used as a data collection tool in the study. The preparation of the Patient Satisfaction Questionnaire was carried out in the following stages.

*First stage*: A literature review and research was conducted on patient expectations and performance criteria.

*Second stage*: The questions planned to be included in the questionnaire were prepared by examining survey studies in the literature.

*Third stage*: The survey questions were evaluated with the participation of 1 academic staff member, 1 senior academic staff member at the upper management level, and 1 administrative staff member from the administrative units of the hospital where the research was conducted, and the questionnaire was finalized. Table 1 shows the Patient Satisfaction Questionnaire.

### Data Collection

The evaluation criteria of the prepared survey questions were established to be evaluated as Disagree (1), Partially Agree (2), No Opinion (3), Good (4), and Very Good (5).

### Data Analysis

The following methods in the IBM SPSS Statistics 20 (IBM SPSS Corp.; Armonk, NY, USA) questionnaire analysis program were used in the analysis of the questionnaires. The following normality tests were performed in the survey study.

1. Examination of Skewness and Kurtosis values in normality tests.
2. Skewness and Kurtosis values divided by the standard error.
3. Kolmogorov–Smirnov and Shapiro–Wilk tests.

Since all the results of the normality tests did not meet the normal distribution conditions, parametric tests were not performed, and the corresponding nonparametric tests, namely Mann–Whitney *U* and Kruskal–Wallis tests, were applied.

## Results

The questionnaire study on whether there is a difference between demographic structures in determining quality perception and patient expectations in health institutions was examined in 5 areas according to 5 demographic characteristics.

When a general evaluation of the questionnaire results is made, as seen in Table 3, the highest average is question 23, with a value of 4.128. In contrast, the lowest average is question 17, with a value of 2.660.

## Statistical Analyses and Results According to Demographic Structures

### Normality Tests

#### Skewness and Kurtosis Test

The range of −2 to 2 was taken as a reference for Skewness and Kurtosis values.

There are specific cut-off points in the literature regarding the Skewness and Kurtosis coefficients to show the normality of the distribution. Accordingly, it is stated that if the distribution is normal, the Skewness and Kurtosis coefficients should be in the range of −1 to 1. If the Skewness coefficient is in the range of −1 to 1, the Kurtosis coefficient can be in the range of −2 to 2, and if the Kurtosis coefficient is in the range of −1 to 1, the Skewness coefficient can be in the range of −2 to 2.5.^7^

As can be seen in Table 4, the Skewness and Kurtosis test values for gender, age, education, occupation, and monthly income categories are between −2 and 2 values, so the data conform to normal distribution.

#### Skewness and Kurtosis Values Divided by Standard Error

“The Skewness and Kurtosis values divided by the Standard Error should be within −1.96 and 1.96.”^7^

As can be seen in Table 4, in the categories of gender, age, education, occupation, and monthly income, the values of Skewness and Kurtosis values divided by the standard error are −1.96 and 1.96 in some categories. In contrast, in some categories, they are not suitable for normal distribution. For this reason, it is understood that the data distribution is unsuitable for normal distribution.

#### Kolmogorov–Smirnov Test

The Kolmogorov–Smirnov test, widely used in the SPSS program and commonly accepted in the literature, determines whether the data set obtained from the sample to which the questionnaires are applied has a normal distribution with the strictest approach. A significance value (asymp. sig.) of *P* < .05 in the result of the Kolmogorov–Smirnov test means that the data are not normally distributed, while a test result of *P* > .05 means that the data are normally distributed.^8^

As seen in Table 4, in the categories of gender, age, education, occupation, and monthly income, according to the Kolmogorov–Smirnov test results, it was seen that some of the values were by the normal distribution since *P* > .05 in some categories, while some values were not by the normal distribution since *P* < .05. For this reason, it is understood that the data distribution is unsuitable for normal distribution.

### Nonparametric Tests

In hypothesis testing, especially in cases where the normality assumption is not met, tests that require very few or no assumptions have been developed. These tests are nonparametric. These tests include nonparametric methods that require almost no assumptions other than that the populations are continuous and that we are not interested in population parameters.^9^

### Mann–Whitney *U-*Test

The Mann–Whitney *U-*test is used to test the null hypothesis stating that “2 independent samples come from the same population” without having to assume that the populations from which the samples were drawn conform to normal distributions.^10^

Since the data in the gender category did not conform to the normal distribution, the Mann–Whitney *U* and test statistics, which are nonparametric tests, were applied.

The results of these tests are shown in Table 5. According to this test result, gender significantly affects quality perception and service expectations at the 5% error level.

### Kruskal–Wallis and Test Statistics

Kruskal–Wallis and test statistics were applied to compare more than 2 independent variables. The results are shown in Table 6.

The reason for choosing this analysis is that it is a technique used to test the significance of the difference between the means of 3 or more groups in groups that do not show normal distribution.^11^

#### Kruskal–Wallis and Test Statistics by Age Category

Age significantly affects quality perception and service expectations at the 5% error level.

#### Kruskal–Wallis and Test Statistics by Educational Status Category

Educational status significantly affects quality perception and service expectations at a 5% error level.

#### Kruskal–Wallis and Test Statistics by Occupation Category

Profession significantly affects quality perception and service expectations at the 5% error level.

#### Kruskal–Wallis and Test Statistics by Monthly Income Category

Monthly income significantly affects quality perception and service expectations at the 5% error level.

## Discussion

According to gender category, 65.7% of the participants are male and 34.3% are female. According to the survey results, gender data do not conform to a normal distribution for both males and females.

According to age category, 26.4% of the participants are in the 21-30 age range, 21.5% in the 31-40 age range, 27.8% in the 41-50 age range, 18.9% in the 51-60 age range, and 5.4% are 61 and above. In the age category, the survey data for 21-30, 31-40, and 51-60 do not conform to a normal distribution, while the data for 41-50 and 61 and above conform to a normal distribution.

According to the education level category, the participants are: 3.8% are primary school graduates, 5.4% middle school and high school graduates, 40.4% college graduates, 15% bachelor’s degree graduates, 24.8% master’s degree graduates, and 10.7% are doctoral graduates. Regarding education, the survey data for middle school and high school graduates follow a normal distribution, while the data for other education groups do not follow a normal distribution.

According to the occupational category, 4.7% of the participants are unemployed, 15% are students, 35.5% are civil servants, 0.5% are private sector managers, 4% are lamu sector managers, 12.4% are housewives, 10.5% are private sector workers, 12.6% are public sector workers, and 4.8% are self-employed. The data for housewives and self-employed workers follow a normal distribution, while the data for other occupational groups do not follow a normal distribution.

According to the monthly income category, the participants are: 15.4% earn less than minimum wage, 7.7% earn minimum wage, 18.5% earn between minimum wage and 6000 TL, 25.9% earn between 6001 and 10 000 TL, and 32.5% earn between 10 001 TL and above. The data of those earning less than minimum wage and those earning at minimum wage level are in line with normal distribution, while the data of other income groups do not conform to normal distribution.

When a general evaluation of the survey results was made in the statistical analysis, it was determined that the average of the survey results was 3.4047. The participants gave the highest evaluation to the question “Did you find the personal care, clothing, and cleanliness of the physician who provided your treatment sufficient?” with an average of 4.1276, and the lowest evaluation to the question “Did you find the cleanliness of the polyclinic and other units where you were examined sufficient?” with an average of 2.6604.

This survey was limited to the hospital, and since it was impossible to survey all patients, it was conducted voluntarily.

It was determined that the survey questions were too many, and the respondents gave random answers due to a lack of knowledge about some questions.

For the article, multiple comparison tests related to demographic structures were conducted, but to comply with the journal format limitation, they could not be included in the article.

Analyzing the questionnaire evaluation based on questions and informing the organization’s managers to take the necessary corrective actions regarding the questions with values below 3.50 will ensure that this study achieves its purpose.

After the corrective actions are taken, it is necessary to verify whether the activities carried out with a questionnaire study covering only these questions have achieved their purpose.

A more comprehensive study can be conducted by dividing the evaluation of the questionnaire questions into categories such as physical, environmental, processes, and management rather than conducting it in general.

## Figures and Tables

**Table 1.** Patient Satisfaction Questionnaire

ATATÜRK UNIVERSITY FACULTY OF DENTISTRY PATIENT SATISFACTION QUESTIONNAIRE						
Your realistic answers to the questions below are essential for our center, which aims to provide quality service. You are expected to express your thoughts on the given sentences by indicating the option that comes closest to you among the expressions of **INADEQUATE—MEDIUM—NO IDEA—GOOD—VERY GOOD**. We thank you in advance for your interest and assistance and wish you a speedy recovery.						
**Personal Information**						
**Gender**	**:**	**( ) Male ( ) Female**				
**Age**	**:**	**( ) 16-20 ( ) 21-30 ( ) 31-40 ** **( ) 40-50 ( ) 50-60 ( ) 60- and above **				
**Educational status**	**:**	**( ) Literate ( ) Primary and secondary education graduate** **( ) High school graduate ( ) Associate degree** **( ) Bachelor**’**s degree ( ) Master**’**s degree/doctorate degree**				
**Occupation**	**:**	**( ) Unemployed ( ) Student ( ) Civil servant** **( ) Private sector manager ( ) Public sector manager ( ) Housewife** **( ) Private sector worker ( ) Public sector worker ( ) Self-employed**				
**Monthly income (TL)**	**:**	**( ) < Minimum wage ( ) Minimum wage ( ) Minimum wage—6000-TL** **( ) 6001-TL-10 0000-TL ( ) 10 001-TL and above**				
	**Please indicate your views on the following statements by checking the appropriate box (x).**	**INADEQUATE**	**MEDIUM**	**NO IDEA**	**GOOD**	**VERY GOOD**
		**1**	**2**	**3**	**4**	**5**
1	Would you evaluate the switchboard service when making an appointment by phone?					
2	What do you think about the online appointment booking system’s usefulness and ease of use?					
3	What do you think about the opportunity to be examined without an appointment?					
4	How would you rate the reception, counseling, and guidance services?					
5	Do you find the opportunity for disabled and elderly patients to receive priority service sufficiently?					
6	Was the sensitivity in choosing the physician you were examined by yourself sufficient?					
7	How would you evaluate our hospital in terms of being able to contact the relevant authorities with your problems and receiving timely answers?					
8	Did you find our hospital adequate in terms of being able to be examined whenever you want?					
9	Did you find the interest and solution-seeking efforts of the administrators to whom you conveyed your problems sufficiently?					
10	What do you think about the hospital parking lot?					
11	How would you evaluate your first impression of the adequacy of the physical condition of our hospital?					
12	Do you think the direction signs and signboards used to facilitate clinic access were adequate?					
13	Were the special elevators and disabled access roads adequate for disabled and elderly patients?					
14	Do you think the cleanliness of the waiting rooms was adequate?					
15	Do you think the number of seats and free spaces in the waiting rooms was adequate?					
16	What do you think about the number and cleanliness of the toilets?					
17	Did you find the cleanliness of the outpatient clinics and other units where you were examined as adequate?					
Table 1.Patient Satisfaction Questionnaire (Continued)						
18	Would you evaluate the number and cleanliness of the elevators used between floors?					
19	Was the visual attractiveness of the materials used to promote the services adequate?					
20	Did you find the number and cleanliness of the devices and instruments in outpatient clinics and other treatment units adequate?					
21	How would you rate the interest and attitude of the staff in the patient registration process towards you?					
22	How was the adequacy of the staff in the patient registration process in answering your questions and providing information?					
23	Did you find the personal care, clothing, and cleanliness of the physician providing your examination adequate?					
24	Was your physician’s interest in you during and after treatment adequate?					
25	Did you find the information provided by your physician about your disease and the treatment process adequate?					
26	How would you rate your physician’s ability to communicate with you in a way you could understand?					
27	Was the nurses’ and staff’s personal care, clothing, and cleanliness adequate?					
28	Was the ability of nurses and other staff to communicate with you in a way you could understand adequate?					
29	Was the sense of trust you felt while receiving medical care adequate?					
ANY THOUGHTS YOU WOULD LIKE TO ADD:						

**Table 2.** Distribution of the Survey Sample According to Demographic Structures

Demographic Structures	Number of People	%
Gender		
Male	281	0.657
Female	147	0.343
Age		
21-30	113	0.264
31-40	92	0.215
41-50	119	0.278
51-60	81	0.189
61 and above	23	0.054
Education status		
Literate	16	0.037
Primary and secondary education graduate	23	0.054
High school graduate	173	0.404
Associate degree	64	0.150
Bachelor’s degree	106	0.248
Master’s degree/doctorate degree	46	0.107
		
Job		
Unemployed	20	0.047
Student	64	0.150
Officer private sector	152	0.355
Private sector manager	2	0.005
Public sector manager	17	0.040
Housewife	53	0.124
Private sector worker	45	0.105
Public sector worker	54	0.126
Self-employment	21	0.048
Monthly income		
<Minimum wage	66	0.154
Minimum wage	33	0.077
Minimum wage—6000-TL	79	0.185
6001-TL to 10 000-TL	111	0.259

**Table 3.** General Statistics of Questionnaire Questions

Cronbach’s Alpha					0.903
Cronbach’s Alpha Based on Standardized Items					0.903
Question	Average	Standard Deviation	Question	Average	Standard Deviation
Q1	3.1495	1.15175	Q16	3.8364	1.15391
Q2	3.1075	1.24047	Q17	2.6799	1.45916
Q3	3.5654	1.25931	Q18	3.3902	1.23917
Q4	3.3435	1.23807	Q19	3.3692	1.23917
Q5	3.4276	1.23261	Q20	3.8364	1.09132
Q6	3.3715	1.23941	Q21	3.1519	1.32454
Q7	3.6472	1.30368	Q22	3.7196	1.20339
Q8	3.4509	1.27668	Q23	4.1472	1.02398
Q9	3.1916	1.40948	Q24	3.1308	1.43123
Q10	3.3248	1.40579	Q25	4.0257	1.01131
Q11	2.9393	1.35018	Q26	3.1636	1.31693
Q12	3.5584	1.17700	Q27	3.3902	1.20094
Q13	3.3785	1.17016	Q28	3.3762	1.14769
Q14	3.0210	1.46690	Q29	3.4533	1.28386
Q15	2.4322	1.32269	Average	3.3793	

**Table 4.** Test of Normality

Category	Statistics		Standard Error	Skewness–Kurtosis Test	Standard Error Test	Kolmogorov−Smirnov Test
Gender						
Male						
Skewness	0.001		0.145	0.001*	0.006*	*P* = .000**
Kurtosis	−0.907		0.290	−0.907*	−3.127**	
Female						
Skewness	−0.317		0.200	−0.317*	**−**1.585*	*P* = .030**
Kurtosis	−0.315		0.397	−0.315*	−0.793*	
Age						
21-30						
Skewness	−0.186		0.227	−0.186*	−0.819*	*P* = .000**
Kurtosis	−0.991		0.451	−0.991*	−2.197**	
31-40						
Skewness	−0.323		0.251	−0.323*	−1.286*	*P* = .000**
Kurtosis	−0.627		0.498	−0.627*	−1.259*	
41-50						
Skewness	−0.107		0.222	−0.107*	−0.481*	*P* = .099*
Kurtosis	−0.045		0.440	−0.045*	−0.000*	
51-60						
Skewness	0.399		0.267	0.399*	0.592*	*P* = .001**
Kurtosis	−1.174		0.529	−1.174*	−1.765*	
61—Yukarisi						
Skewness	−0.374		0.481	−0.374*	1.494*	*P* =.092*
Kurtosis	−0.077		0.935	0.077*	−0.082*	
Educational status						
Literate						
Skewness	−1.229		0.564	−1.229*	−2.179**	*P* = .000**
Kurtosis	−0.266		1.091	−0.266*	−0.243*	
Primary and secondary education graduate						
Skewness	−0.123	0.481		−0.123*	−0.255*	*P* = .200*
Kurtosis	−0.614	0.935		−0.614*	−0.656*	
High school graduate						
Skewness	−0.084	0.185		−0.084*	−.454*	*P* = .000**
Kurtosis	−1.010	0.367		−1.010*	−2.750**	
College graduate						
Skewness	−0.041	0.299		−0.041*	−0.137*	*P* = .029**
Kurtosis	−0.609	0.590		−0.609*	−1.032*	
Undergraduate degree						
Skewness	0.027	0.235		0.027*	0.114*	*P* = .049**
Kurtosis	−0.837	0.465		−0.837*	−1.800*	
Master/Ph.D. graduate						
Skewness	−0.195	0.350		−0.195*	−0.557*	*P* = .001**
Kurtosis	−1.229	0.688		−1.229*	−1.786*	
Job						
Unemployed						
Skewness	0.422	0.512		0.422*	0.824*	*P* = .010**
Kurtosis	−0.780	0.992		−0.780*	−0.786*	
Student						
Skewness	0.213	0.299		0.213*	0.712*	*P* = .000**
Kurtosis	−1.290	0.590		−1.290*	−2.186**	
Officer						
Skewness	0.361	0.197		0.361*	1.832*	*P* = .000**
Kurtosis	−0.784	0.391		−0.784*	−2.005**	
Private sector manager						
Skewness	0	0		0	0	***P*** = .000**
Kurtosis	0	0		0	0	
Public sector manager						
Skewness	0.749	0.550		0.749*	1.361*	*P* = .003**
Kurtosis	−0.654	1.063		−0.654*	−0.615*	
Housewife						
Skewness	0.161	0.327		0.161*	−0.492*	*P* = .537*
Kurtosis	0.002	0.644		0.002*	0.003*	
Private sector worker						
Skewness	−0.600	0.354		−0.600*	−1.694*	*P* = .021**
Kurtosis	0.263	0.695		0.263*	0.378*	
Public sector worker						
Skewness	−0.308	0.325		−0.308*	−0.947*	*P* = .002**
Kurtosis	−1.069	0.639		−1.069*	−1.672*	
Self-employment						
Skewness	0.175	0.501		0.175*	0.349*	*P* = .556*
Kurtosis	1.135	0.972		1.135*	1.167*	
Monthly income						
<Minimum wage						
Skewness	−0.120	0.295		−0.120*	−0.406*	*P* = .200*
Kurtosis	0.081	0.582		0.081*	0.139*	
Minimum wage						
Skewness	0.252	0.409		0.252*	0.616*	*P* = .200*
Kurtosis	−0.196	0.798		−0.196*	−0.245*	
Minimum wage-6000-TL						
Skewness	−0.350	0.271		−0.350*	−1.291*	*P* = .000**
Kurtosis	−0.876	0.535		−0.876*	−1.637*	
6001-TL-10 000-TL						
Skewness	−0.251	0.229		−0.251*	−1.096*	*P* = .002**
Kurtosis	−0.855	0.455		−0.855*	−1.879*	
10 001-TL and above						
Skewness	0.387	0.206		0.387*	1.878*	*P* = .020**
Kurtosis	−0.743	0.408		−0.743*	−1.821*	

*Data conform to normal distribution.

**Data do not conform to normal distribution.

**Table 5.** Mann–Whitney *U-*Test

Mann–Whitney *U-*Test				Test Statistics			
	N	Mean Rank	Sum Of Ranks	Mann–Whitney *U*	Wilcoxon W	*Z*	Asymptotic Significance (2-tailed)
Gender							
Male	281	205.93	57865.00	18244.000	57865.000	−1.987	*P *= .047*
Female	147	230.89	33941.00				

*Since *P* < .05, there is a significant difference between the groups.

**Table 6.** Kruskal–Wallis Test

Kruskal–Wallis Test			Test statistics		
	N	Mean Rank	Chi-Square	*df*	Asymptotic Significance
Age					
21-30	113	225.82	11.393	4	0.022*
31-40	92	227.20			
41-50	119	219.52			
51-60	81	173.22			
61 and above	23	227.48			
Educational status					
Literate	16	266.56	19.299	5	0.002*
Primary and secondary education graduate	23	267.87			
High school graduate	173	189.8			
College graduate	64	201.24			
Undergraduate degree	106	233.23			
Master/Ph.D. graduate	46	239.46			
Job					
Unemployed	20	301.20	52.840	8	0.000*
Student	64	184.87			
Officer	152	189.89			
Private sector manager	2	371.50			
Public sector manager	17	179.09			
Housewife	53	243.79			
Private sector worker	45	267.00			
Public sector worker	54	180.88			
Self-employment	21	314.07			
Monthly income					
Minimum wage	66	265.21	19.518	4	0.001*
Minimum wage	33	225.39			
Minimum wage-6000- TL	79	214.70			
6001-TL-10 000-TL	111	218.25			
10 001-TL and above	139	184.73			

*Since *P* < .05, there is a significant difference between the groups.

## Data Availability

The data that support the findings of this study are available on request from the corresponding author.

## References

[1] Çavmak Ş Çavmak D . Historical development of health services in Turkey and conversion program in health. *Sağlık Yönetimi Dergisi. * 2017;1(1):48 57.

[2] General Directorate of Health Services. Department of quality, accreditation and employee rights in healthcare. ProMed-Mail. 23.09.2024. Available at: https://kalite.saglik.gov.tr/TR-9216/saglikta-kalite-degerlendirmeleri.html.

[3] Aydemir FA . The Effect of Total Quality Management on Business Life and Its Implementation in Health Sector. [Selçuk University Institute of Social Sciences, Master’s Thesis]. 2006.

[4] Kayral İH . Perceived Service Quality in Health Enterprises and Research According to Hospital Types in Ankara. [Gazi University Institute of Social Sciences, Doctoral Thesis]. 2012.

[5] Yıldız S Yıldız SE . The effect of service quality on customer satisfaction: A research in state and university hospitals in Kars. Infor Econ Manag. 2011;6(2):125 140.

[6] Büyüköztürk Ş . Data Analysis for Social Sciences. 30th ed. Ankara: Pegem; 2011.

[7] Uysal İ Kılıç A . Normal distribution dilemma. Anadolu J Educ Sci Int. 2022;12(1):220 248. (10.18039/ajesi.962653).

[8] Erdil M . A Study on Customer Oriented Management in Construction Sector. [Adana Alparslan Türkeş Science and Technology University Institute of Graduate Education, Master’s Thesis]. 2022.

[9] Eltas Ö . Mann-Whitney U in small samples used in biostatistics studies. Atatürk Univ Vet Sci. 2021;16(1):88 94.

[10] Bindak R . Comparison of Mann-Whitney U and Student’s t-test regarding Type I Error and Power: Monte Carlo Simulation study. Afyon Kocatepe Univ Sci Eng. 2014;14(1):5 11.

[11] Çetinkaya B . An Application on Behavioral Economics: the Effect of Music on Consumption. [Bolu Abant Izzet Baysal University Institute of Social Sciences Master’s Thesis]. 2019.

